# Periodontal Inflamed Surface Area (PISA) associates with composites of salivary cytokines

**DOI:** 10.1371/journal.pone.0280333

**Published:** 2023-02-15

**Authors:** Vera Tang, Bubak Hamidi, Malvin N. Janal, Cheryl A. Barber, Benjamin Godder, Leena Palomo, Angela R. Kamer

**Affiliations:** 1 Department of Periodontology and Implant Dentistry, College of Dentistry, New York University, New York, New York, United States of America; 2 Department of Epidemiology and Health Promotion, College of Dentistry, New York University, New York, New York, United States of America; 3 Department of Basic Sciences and Craniofacial Biology, College of Dentistry, New York University, New York, New York, United States of America; 4 Cariology and Comprehensive Care, College of Dentistry, New York University, New York, New York, United States of America; All India Institute of Medical Sciences, INDIA

## Abstract

**Background:**

Periodontal disease (PerioD) is a chronic, complex inflammatory condition resulting from the interaction between subgingival dysbiotic bacteria and the host immune response leading to local inflammation. Since periodontal inflammation is characterized by multiple cytokines effects we investigated whether Periodontal Inflamed Surface Area (PISA), a continuous measure of clinical periodontal inflammation is a predictor of composite indexes of salivary cytokines.

**Methods and findings:**

In a cross-sectional study of 67 healthy, well-educated individuals, we evaluated PISA and several cytokines expressed in whole stimulated saliva. Two salivary cytokine indexes were constructed using weighted and unweighted approaches based on a Principal Component Analysis [named Cytokine Component Index (CCI)] or averaging the (standardized) level of all cytokines [named Composite Inflammatory Index (CII)]. In regression analysis we found that PISA scores were significantly associated with both salivary cytokine constructs, (CCI: part R = 0.51, p<0.001; CII: part R = 0.40, p = 0.001) independent of age, gender and BMI showing that single scores summarizing salivary cytokines correlated with severity of clinical periodontal inflammation.

**Conclusions:**

Clinical periodontal inflammation may be reflected by a single score encompassing several salivary cytokines. These results are consistent with the complexity of interactions characterizing periodontal disease. In addition, Type I error is likely to be avoided.

## Introduction

Periodontal disease (PerioD) is a chronic, inflammatory condition present in more than 50% of the population [1]. It results from the interaction between subgingival dysbiotic bacteria and the host immune response [2–4] leading to local inflammation characterized by tissue infiltration with immune cells and high pro-inflammatory cytokines such as IL-8, IL-1β, IL-6 and TNFα [5]. While periodontal disease can be quantified using a combination of multiple measures such as bleeding on probing (BOP), tissue color, contour, and pocket depth, clinical attachment loss, inflammatory quantification using a single continuous index is desirable. Periodontal Inflamed Surface Area (PISA) is such an index and has been described and validated [6,7].

The significance of local periodontal inflammation resides not only in its disease maintenance and contribution to its progression [8,9] but also in its systemic impact including CVD [10], kidney [11], diabetes [12,13], Alzheimer disease [14] and rheumatoid arthritis [15]. Proposed biological mechanisms for these systemic effects include indirect and direct pathways: inflammatory cytokines [14] derived from periodontal tissue would contribute to other systemic diseases indirectly by increasing systemic inflammation or directly by acting on the targeted organ. Studying this mechanism requires direct quantification of cytokines in periodontal tissue that would be highly impractical. Therefore, salivary cytokines could serve as surrogates for periodontal inflammation.

Many studies investigated salivary cytokines in relation to a variety of continuous and discrete measures of periodontal disease [9,16]. However, few studies explored their relationship with PISA [17], the continuous index of clinical periodontal inflammation. The aim of this study was to determine whether there is a relationship between PISA and several salivary cytokines. Multiple comparisons in such a study increase the chance of Type I error (false positives). Therefore, a composite index including all or a few salivary cytokines would circumvent this problem. We tested the hypothesis that these salivary cytokines could be reasonably aggregated and that PISA was associated with these composite scores.

## Material and methods

### Study design and population

This is a cross-sectional study in which the subjects were recruited from an existing cohort participating in studies relating periodontal disease to Alzheimer’s disease [18,19] whose characteristics were described previously [18,19]. This study was approved by the New York University Institutional Review Board. We obtained written informed consent from all subjects. Sixty-seven (67) subjects with both periodontal and salivary cytokine measures were included in this study. *Inclusion Criteria*: All included subjects were required to be ≥ 45 years, with ≥12 years of education and be cognitively unimpaired. *Exclusion Criteria*: Individuals were excluded if they had significant comorbidities, including stroke, diabetes, uncontrolled hypertension, head trauma, any neurodegenerative disease, and chronic depression. Subjects taking anti-inflammatory medications for chronic conditions (i.e. NSAIDS, anti-TNFα), or antibiotics or having periodontal treatment within 3 months of the periodontal evaluation were also excluded.

Salivary cytokines were sampled from whole saliva.

#### Saliva collection

Stimulated whole-saliva samples were collected on ice in a sterile 50 ml plastic conical centrifuge tube. After rinsing with drinking water to clear food debris, the subject chewed on a piece of unflavored gum and spit the saliva in the sterile tube until 5mls were collected. Then, the saliva was centrifuged for 25 minutes at 2,500rpm at 4C. The aliquoted supernatant was stored at -80°C until cytokine analysis.

*Salivary cytokines [Interleukin-1 (IL-1β)*, *IL-6*, *IL-8*, *IL-13 and tumor necrosis factor- α (TNF-α*, *IL-2*, *IL-4*, *INF-gamma)]* levels were assessed in stimulated saliva using electrochemiluminescence method (MSD platform). Human V-Plax proinflammatory panel-Cat# K15049D measuring the above cytokines was performed according to the manufacture protocol. In the final step, after the Read Buffer was added, the signal from the MSD plates was read on the Meso Scale Discovery (MSD) Sector Imager 2400 at 620 nm. Cytokines expressed in pg/ml were quantified using a standard curve of appropriate standards provided by the kit. Each sample was assayed in duplicate and the average was used for analysis. Our analyses excluded cytokines with >10% of samples scoring outside the dynamic range of the standard curve: INF-gamma, IL-2 and IL-4: INF-gamma with 11 subjects (16%), IL-2: 16 subjects (24%), and IL-4: 48 subjects (72%) testing bellow the detection limit.

#### Periodontal exam

Subjects received an oral-periodontal examination, as previously described [18]. Briefly, this exam encompassed examination of 6 surfaces of each tooth for probing depth. (PD), clinical attachment loss (CAL) and bleeding on probing (BOP). Pocket depth measures were assessed at six sites per tooth using a Michigan probe [20] and defined as the linear distance in millimeters from the gingival margin to the base of the periodontal pocket while BOP was assessed at each probing site after the quadrant probing.

Results of the periodontal exam were summarized as the Periodontal Inflamed Surface Area (PISA) scores expressed in mm^2^ [7]. PISA is calculated from PD and BOP using an EXEL sheet available from Nesse’s publication (http://www.parsprototo.info/). Since PD measures the past and present disease and BOP is the most reliable measure for assessing gingival inflammation [21], therefore, combining both measures in a single PISA index is best reflective of current clinical periodontal inflammatory status. Low vs. High PISA denoting low vs. high periodontal inflammation was defined using 450mm^2^ as threshold [22].

Demographic information (age, gender, and education), systemic factors (comorbidities), oral (brushing, flossing, dentist visits) and smoking behaviors were obtained by a standardized examiner-conducted interview at the time of the oral examination [18].

### Statistical analysis

All our analyses were performed using IBM SPSS Statistics software, version 27 (IBM Corp., Armonk, NY). Continuous data are presented as means and standard deviation (SD) and categorical data as percentages. Normality was tested by Kolmogorov-Smirnov and log10 transformation was used to normalize the distributions for each salivary cytokine. Group differences for cytokines were assessed using Students t-test.

To reduce the dimensionality of our data (numbers of cytokines) we used weighted and unweighted approaches based on a Principal Component Analysis (PCA) or averaging the (standardized) level of all cytokines. Thus, we created two salivary cytokine indexes: 1. salivary cytokine component index (CCI) and 2. salivary cytokine composite inflammatory index (CII).

1) Salivary CCI was derived from Principal Component Analysis (PCA) that included 6 salivary cytokines (IL-1β, IL-6, IL-8, IL-10, IL-13 and TNF-α). Principal Component Analysis (PCA) is a wel**l-**known analysis that is based on reducing the dimensionality of data such as multiple-correlated variables into a reduced number of uncorrelated components called Principal Components. PCA has been previously used in cytokine analyses [23,24].

Our data supports the use of PCA: cytokines are presented as continuous data and there was a linear relationship between them. Linearity between all variables was evaluated using a correlation matrix while sampling adequacy was evaluated using the Kaiser-Meyer-Olkin (KMO). The correlation matrix showed that all variables have at least one correlation with another variable greater than the 0.3 cut-off. The overall KMO measure of 0.723 is considered fair. Similarly, for each individual variable the KMO measure ranged between 0.907 and 0.646 considered adequate. Bartlett’s test of sphericity was statistically significant (p<0.0005) indicating that the components of the analysis were correlated. The *a priori* decision was to retain all the components with the eigenvalue greater than unity. An eigenvalue is a measure of the variance accounted for by one component. Component loadings are the correlations between the cytokines and the component.

2) Salivary CII was derived using statistical means. We constructed a salivary CII defined as the statistical mean of the 6 salivary cytokines: IL-1β, IL-6, IL-8, IL-10, IL-13 and TNF-α.

#### Statistical models

Correlations and linear regression models were used to assess PISA relationships with salivary cytokines (individual salivary cytokine, and the two aggregated inflammatory indices). In initial models, we evaluated the association of potential confounders with the cytokines: age, gender, education, BMI, behaviors (smoking, brushing, flossing, dentist visits), systemic conditions (no medical vs. medical condition). Gender and BMI were significant and were included in the multivariate models. Although age was not significant, due to its reported association with both cytokines and PISA, it was also included in the final model.

To determine whether PISA was uniquely associated with salivary cytokines, regression analyses were performed in which either the individual salivary cytokines, the salivary CCI or the salivary CII were predicted from the PISA score and the relevant covariates. The part correlations (part R), unstandardized regression coefficients (β) and the 95% confidence interval (95%CI) of the β were obtained. Statistical significance was set at p<0.05.

## Results

The characteristics of our population are shown in Table 1. Our population was relatively homogeneous. Most were white, elderly and highly educated. Females were slightly more represented. Subjects were relatively healthy with >65% having only one or no medical condition and only 5 (7.5%) were current smokers. Mean BMI was 26.6 (SD = 5.2) ranging from 19 to 48. Most subjects had low PISA accounting for 65.7%. All subjects had CAL≥5mm and therefore they had Stage III and IV periodontitis.

**Table 1:** Characteristics of the study population (n = 67).

Demographic			Min-Max		
Age [Mean (SD)]		67.0 (9.0)	44–89		
Age Categories:					
	≤50 [n (%]	2 (3)			
	51–60	16 (23.9)			
	61–70	24 (35.8)			
	71–80	21 (31.3)			
	>81	4 (6)			
Gender n (%)					
Female		38 (56.7)			
Race n (%)					
	White	64 (95.5)			
Education [Mean (SD)]		17.5 (2.3)	13–24		
Education Categories:					
	≤15 [n (%]	8 (11.9)			
	16–20	54 (80.6)			
	>21	5 (7.5)			
BMI [Mean (SD)]		26.59 (5.24)	19–48		
BMI Categories:					
	≤25 [n (%]	31 (46.3)			
	26–30	24 (35.8)			
	>31	12 (17.9)			
PISA					
	Low (^2^450mm2)	44 (65.7)			
	High (>450mm2)	23 (34.3)			
Cytokine pg/ml	[Mean (SD)]	Range		Median	Log10
IL-1_		40.18 (46.31)	U(1)-274.83	27.05	1.38
IL-6		1.59 (4.19)	0.09–34.16	0.68	-0.12
IL-8		341.14 (168.62)	13.12–738.37	341.61	2.46
IL-10		0.83 (2.76)	0.02–16.39Â	0.19	-0.67
IL-13		2.39 (3.77)	U(4)-28.55	1.45	0.19
TNF-α		1.61 (2.91)	U(1)-16.48	0.71	-0.08
Mean cytokines		64.62	4.48–172.56	63.65	

PISA = Periodontal inflamed surface area.

U = undetectable (the number of samples); Log10 = mean of log10 values.

Mean cytokines = mean of sum of Il-1β, IL6, IL-8, IL10, IL-13, TNF-α.

The levels of salivary cytokines are shown in Table 1. Gender differences were found only for IL-10 [(Female: 1.31 (3.61) vs. Male: 0.20 (0.18), p = 0.05]. There were no differences in cytokine levels as a function of smoking status, medical conditions or various dental hygiene behaviors. IL-6 was higher in those overweight [Mean (SD): 2.42 (5.67) vs. 0.68 (0.67), p = 0.002 (MWU test)]. PISA or salivary cytokines did not correlate with age or years of education.

### PISA associates with salivary inflammatory cytokines

Table 2 shows moderately strong correlations between PISA and all of the salivary cytokines except IL-10. The various cytokines were also moderately to strongly related to one another.

**Table 2:** Correlations between PISA and salivary cytokines (log).

Cytokine	PISA	IL-1β	IL-6	IL-8	IL-10	IL-13
IL-1β	0.40+					
IL-6	0.34***	0.40+				
IL-8	0.27*	0.43+	0.24*			
IL-10	0.07	0.37***	0.34***	0.54+		
IL-13	0.39+	0.44+	0.45+	0.63+	0.53+	
TNFα	0.41+	0.66+	0.45+	0.44+	0.31**	0.61+

* = p≤0.05

** = p≤0.01

*** = p≤0.005; + = p≤0.001 based on Pearson correlations.

Regression analysis showed that after age, gender and BMI adjustment, PISA maintained significant associations with IL-1β (part R = 0.42, p<0.001), IL-6 (part R = 0.40, p = 0.001), IL-8 (part R = 0.33, p = 0.009), IL-13 (part R = 0.44, p<0.001) and TNFα (part R = 0.43, p<0.001).

Thus, the addition of PISA to age, gender and BMI, led to a statistically significant increase in R2 for each cytokine ranging from 10 to 18%.

### PISA associates with salivary CCI

PCA was applied with the goal of reducing the number of salivary cytokines into fewer components. PCA showed only one component with an eigenvalue greater than unity and it explained 52% of the total variance. Table 3 shows component loadings (correlations between the variable and the component). Inspection shows that all cytokines loaded strongly on this component, labeled CCI.

**Table 3:** Cytokine component loading scores.

IL-1β	0.771
IL-6	0.657
IL-8	0.618
IL-10	0.672
IL-13	0.816
TNFα	0.780

**Fig 1:**
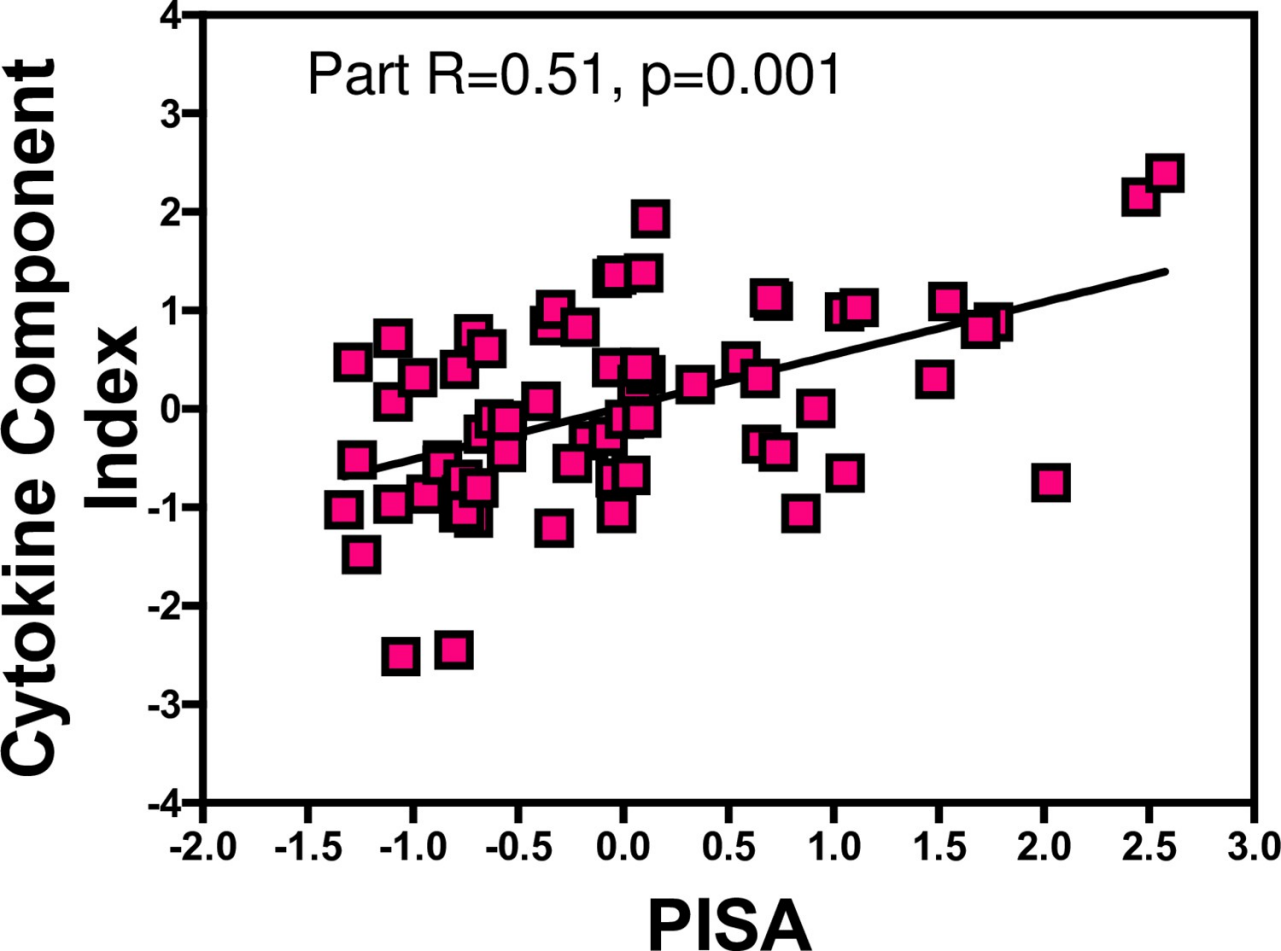
Partial regression plot showing the relationships between PISA and salivary CCI. The x and y axes are the residuals for PISA and CCI adjusted for age, gender and BMI. The partial R and p values are shown.

In regression analyses, the CCI score was predicted by PISA score (part R = 0.51, p<0.001), gender (part R = 0.27, p = 0.03) and BMI (part R = 0.42, p<0.001), yielding a total R2 of 0.37, p<0.001). We re-run the analyses using age, education and BMI categories and the results were similar. Fig 1 shows the partial regression plots of PISA and CCI. There was no interaction between PISA and Gender, BMI or age (Pinteraction>0.05).

### PISA associates with the salivary CII

**Fig 2:**
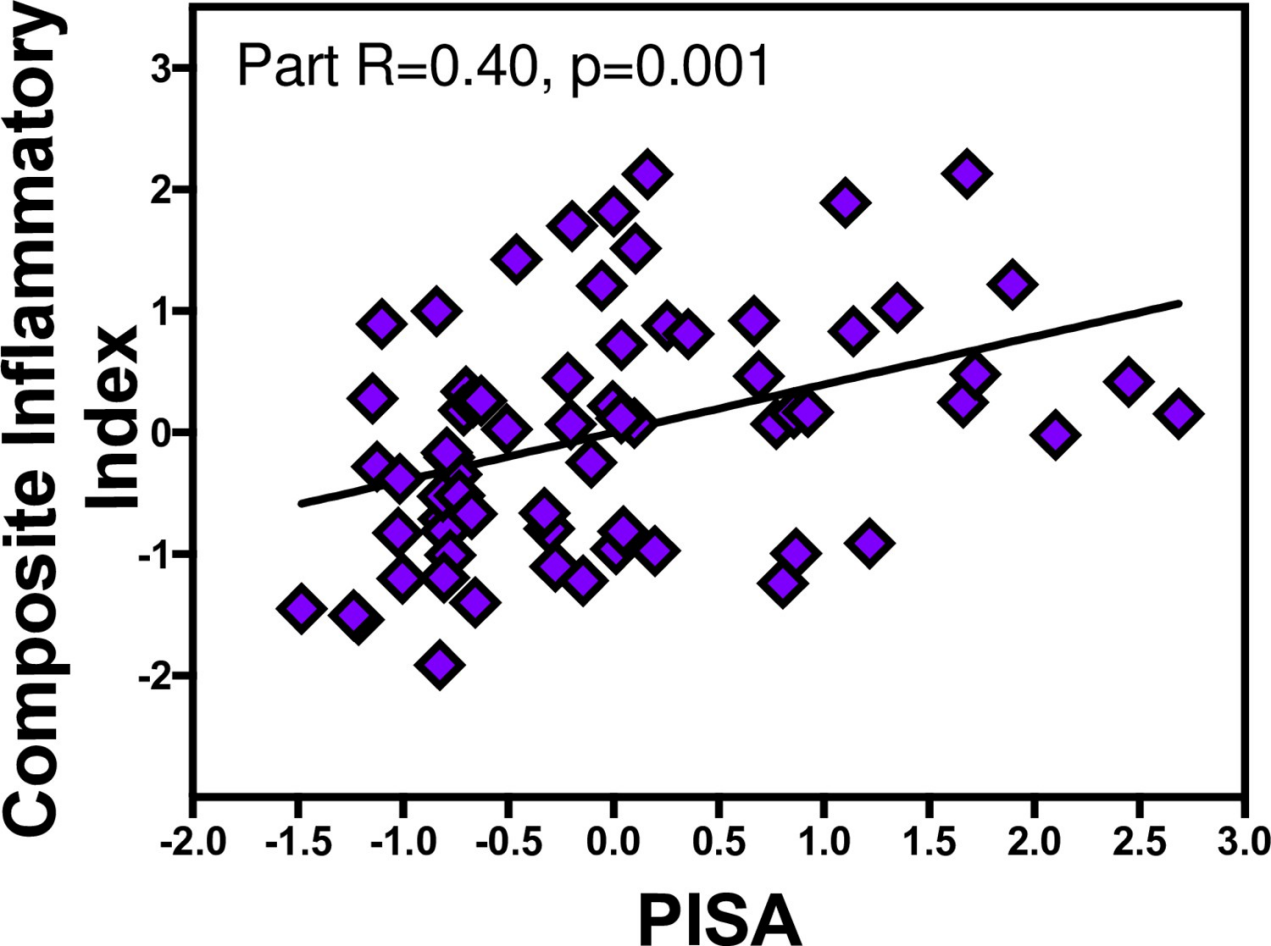
Partial regression plots show the relationships between PISA and salivary CII. The x and y axes are the residuals for PISA and CII adjusted for age, gender and BMI. The partial R and p values are shown.

In regression analyses, the salivary CII score was predicted by PISA score (part R = 0.40, p = 0.001), gender (part R = 0.29, p = 0.02) and BMI (part R = 0.40, p = 0.001), yielding a total R2 of 0.29, (p = 0.001). Re-running the analyses using age, education and BMI categories, resulted in the same outcome. Fig 2 shows the partial regression plots of PISA and salivary composite inflammatory index. Regression analyses are shown in S1 Table. There was no interaction between PISA and Gender, BMI or age (Pinteraction>0.05).

## Discussion

In this study we found that PISA scores were significantly associated with both salivary cytokine constructs, salivary CCI and salivary CII and these associations were independent of age, gender and BMI. Smoking was not significant in the model suggesting that smoking was not a confounder of the relationship between PISA and salivary cytokine indexes. These results support our hypothesis that a single score can be used to summarize levels of salivary cytokines and that this score is highly correlated with the severity of periodontal inflammation. PISA association with salivary CCI was stronger (R2 = 0.37) than its association with salivary CII (R2 = 0.29) because the former index accurately weighted the component scores. However, salivary CII is easy to construct and use and its numerical value is easy to interpret. Periodontal disease results from a complex interaction between periodontal bacteria and immune response supporting multiple cytokines as predictors of PISA. Our population had stage III and IV periodontitis. Therefore, it is unknown if this relationship would be similar within or between other stages of periodontitis and health.

PISA as assessed by PD and BOP represents the overall clinical periodontal inflammation. On the other hand, salivary cytokines are a correlate of the molecular make-up of the oral environment. Periodontal inflammation is best represented by the presence of inflammatory cytokines in gingival crevicular fluid (GCF) the proximal periodontal fluid. However, sampling of GCF poses challenges: it is difficult and time consuming; it reflects gingival inflammation at a specific site and therefore numerous sites must be sampled [3]; sampling yields small amounts and GCF can easily be contaminated [4,5]. Therefore, salivary cytokines offer an alternative and advantages: the sampling is relatively easy, enough sample can be collected and reflects aggregate inflammation. Here we showed that periodontal inflammation contributes to the cytokine burden assayed from whole saliva. Salivary cytokines were also associated with BMI, a finding consistent with salivary cytokines reflecting systemic conditions [25,26].

Several studies have shown that PISA is associated with several systemic diseases including CVD [27], dementia [28], rheumatoid arthritis [29] kidney disease [30], diabetes [31]. PISA has also been found to be associated with plasma CRP [30,32], a measure of systemic inflammation. One plausible mechanism linking PISA to systemic diseases is via systemic inflammation triggered by periodontal disease derived cytokines. Our study supports this possibility. We showed that salivary cytokines are reflecting the severity of periodontal inflammation and therefore is plausible that they contribute to elevated systemic CRP. Testing this hypothesis would require measures of plasma CRP.

We used two salivary cytokine indexes and both included the cytokines IL-1β, IL-6, IL-8 and TNF-α. These are well known pro-inflammatory cytokines and their association with periodontal inflammation is not surprising. In fact, numerous studies investigating the levels of these cytokines in association with periodontal disease found positive results [33] although there are also contradictory reports [34]. The reasons for discordant results could be multiple including the nature of the population, the measures of periodontal disease, the methodology assessing the cytokines, the nature of the saliva (whole, stimulated vs. unstimulated). Salivary IL-1β has been consistently found to be elevated in subjects with periodontal disease [35–37]. IL-8 is a proinflammatory cytokine with significant roles in chemotaxis and migration of various cells and elevated with increased pocket depth [38]. Among the inflammatory molecules most studies examined IL-1β [36], IL-6 [16], and TNF-α [39] and few investigated IL-10 [16]. Our study also assessed the concomitant expression of the cytokines IL-10 and IL-13 [40].

IL-10 is an anti-inflammatory cytokine with regulatory immune functions. In periodontitis it has protective functions and inhibits pro-inflammatory cytokine production. In our study, IL-10 loaded on the PCA inflammatory component in tandem with the other pro-inflammatory molecules. Its contribution to the inflammatory component was not anticipated. However, it can be explained by its up-regulation in response to an increased inflammatory milieu. Increased inflammation may trigger a feedback mechanism to counteract further inflammation. In addition, IL-10 could have a proinflammatory role [41].

Interestingly, IL-13 loading on CCI was the strongest. IL-13 is a pleomorphic cytokine with both anti-inflammatory and proinflammatory functions. In its role as anti-inflammatory cytokine, IL-13 inhibits the secretion of pro-inflammatory cytokines including TNF-α, IL-1β, IL−6, and IL−8 [42,43]. However, in certain conditions, IL-13 can lead to enhanced inflammation [44]. Il-13 can have toxic effects on the epithelial cells [45,46]. Similarly, in periodontitis IL-13 can have anti-inflammatory [47] and proinflammatory effects [48]. In experimental gingivitis, IL-13 was one of the cytokines associated with gingival inflammation and its fast development [49]. Significantly, IL-13 stimulates B-lymphocytes and induces the synthesis of immunoglobulins [50]. Chronic periodontitis is characterized by heavy infiltration with B-lymphocytes and these are important effector cells mediating inflammation and destruction in periodontitis [51–53] and thus could explain the strong IL-13 loading.

### Strengths and weaknesses

Several strengths characterized our study. Our sample was quite homogeneous consisting of elderly, well-educated and relatively healthy individuals. Periodontal measures and saliva collection were standardized and salivary cytokine assays were determined blindly to the periodontal assessment.

There are several limitations related to our study that include the design, population characteristics, and sample size. As a cross-sectional study, it shows only a correlate of periodontal inflammation to salivary cytokines. The number of subjects was moderate. An additional weakness was the periodontal status of our population: all had severe periodontitis with various degree of clinical periodontal inflammation. The ranges of PISA in our study: mild-moderate range with few severe forms. Due to their homogeneity, and limitations described these results may not apply to general population. It is desirable to test our hypothesis in a diverse population composed of periodontically healthy subjects, gingivitis and various stages and grades of periodontitis.

In conclusion, we showed that two salivary cytokine indices could be used to better characterize the inflammatory consequences of periodontal inflammation. However, subsequent larger studies should be done to validate these indices in a heterogeneous population. Studies that manipulate periodontal inflammation are also needed to support a causal link with cytokine responses.

## Supporting information

S1 Table Multiple regression analysis predicting salivary cytokine indexes. (XLSX)
